# Supplementary material for: Pregnancy-Specific Beta-1-Glycoprotein 1 Increases HTR-8/SVneo Cell Migration through the Orai1/Akt Signaling Pathway
Source: Biomolecules. 2024 Feb 29;14(3):293. doi: 10.3390/biom14030293 (PMC10968057; doi:10.3390/biom14030293)

Original western blot 1

Orai1 expression was expressed in HTR/8-Svneo cultured in 0, 0.1 or 0.5 μg/mL pregnancy-specific beta-1-glycoprotein 1 (PSG1) medium for 24 h.

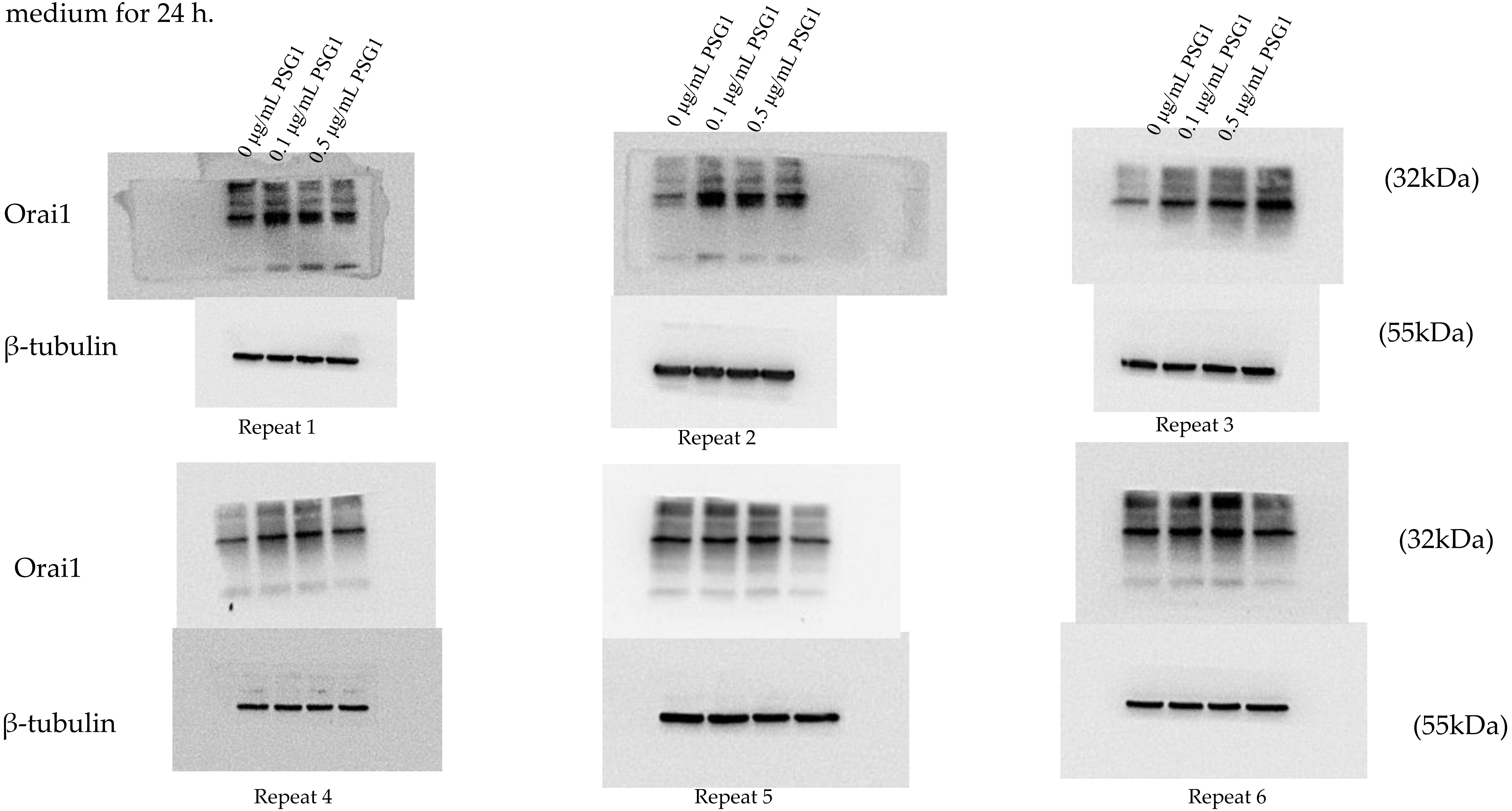

Original western blot 2

Akt/p-Akt expression was expressed in HTR/8-SVneo cells cultured in 10 μM MRS1845 and/or 0.1 μg/mL ZSTK474 and/or 0.1 μg/mL PSG1 medium for 24 h.

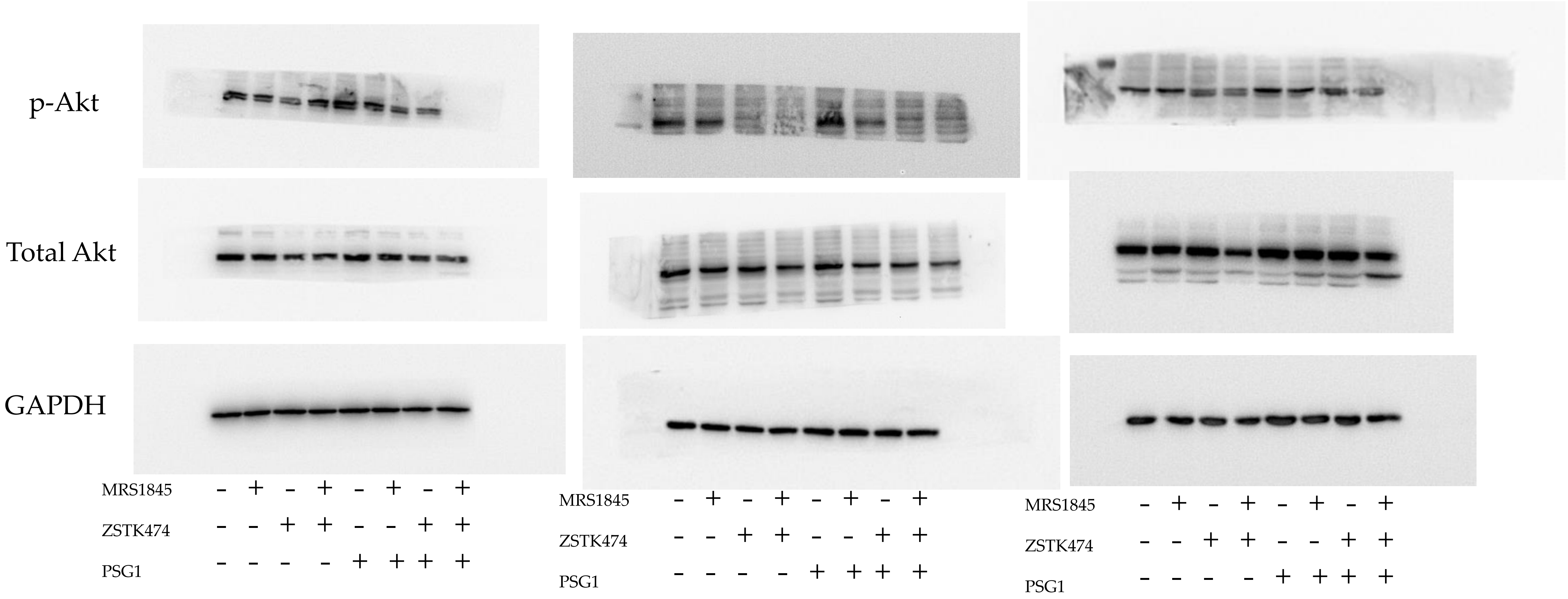

Supplement: Supplementary file 1 [file biomolecules-14-00293-s001.zip › biomolecules-2797733-supplementary.pdf]
